# Finding cannabinoids in hair does not prove cannabis consumption

**DOI:** 10.1038/srep14906

**Published:** 2015-10-07

**Authors:** Bjoern Moosmann, Nadine Roth, Volker Auwärter

**Affiliations:** 1 Institute of Forensic Medicine, Forensic Toxicology, Medical Center - University of Freiburg, Albertstr. 9, 79104 Freiburg, Germany

## Abstract

Hair analysis for cannabinoids is extensively applied in workplace drug testing and in child protection cases, although valid data on incorporation of the main analytical targets, ∆9-tetrahydrocannabinol (THC) and 11-*nor*-9-carboxy-THC (THC-COOH), into human hair is widely missing. Furthermore, ∆9-tetrahydrocannabinolic acid A (THCA-A), the biogenetic precursor of THC, is found in the hair of persons who solely handled cannabis material. In the light of the serious consequences of positive test results the mechanisms of drug incorporation into hair urgently need scientific evaluation. Here we show that neither THC nor THCA-A are incorporated into human hair in relevant amounts after systemic uptake. THC-COOH, which is considered an incontestable proof of THC uptake according to the current scientific doctrine, was found in hair, but was also present in older hair segments, which already grew before the oral THC intake and in sebum/sweat samples. Our studies show that all three cannabinoids can be present in hair of non-consuming individuals because of transfer through cannabis consumers, via their hands, their sebum/sweat, or cannabis smoke. This is of concern for e.g. child-custody cases as cannabinoid findings in a child’s hair may be caused by close contact to cannabis consumers rather than by inhalation of side-stream smoke.

Among illicit drugs cannabis is still the drug showing the highest prevalence, with an estimated 125–227 million consumers worldwide^1^. In hair analysis, the two main targets for cannabinoid analysis are the psychoactive Δ9-tetrahydrocannabinol (THC) and its metabolite 11-*nor*-9-carboxy-Δ9-tetrahydrocannabinol (THC-COOH)^2^. Typical models for incorporation of drugs into hair include passive diffusion from blood capillaries into matrix cells at the basement membrane of the hair follicle and diffusion from sweat or sebum into the completed hair shaft, but also the possibility of external contamination is an issue^2^,^3^. While presence of THC-COOH, which is only formed inside the body^4^, is considered a proof of ingestion/consumption according to the current scientific doctrine when detected in hair^5^,^6^,^7^,^8^, analysis for THC alone is still common laboratory practice, because THC-COOH hair concentrations are extremely low and afford the use of expensive instrumentation^9^,^10^. However, due to THC being present in cannabis smoke, there is a high probability of biased results caused by external contamination of the hair^3^,^11^, and the mechanism of incorporation for THC-COOH is still unknown.

Recently, in addition to THC, relatively high Δ9-tetrahydrocannabinolic acid A (THCA-A) concentrations were detected in forensic hair samples^12^,^13^. THCA-A is the non-psychoactive biosynthetic precursor of THC and the main cannabinoid in fresh cannabis plant material. When heated, e.g. during smoking or baking, THCA-A is decarboxylated yielding THC^14^ (Fig. 1). As relevant incorporation through the bloodstream could not be verified in previous investigations, the major part of this cannabinoid seems to originate from handling of cannabis material and subsequent transfer to the hair^15^. Furthermore, the chemical instability of THCA-A entails the risk of artifactually elevating the THC concentration during the analytical process, potentially leading to false positive findings^12^,^13^.

In this article, two studies are described in order to elucidate the main routes of incorporation for THC, THC-COOH and THCA-A into human hair and to provide a valid basis for correct interpretation of hair analysis results.

## Results

### Oral intake of THCA-A

To definitely exclude a relevant incorporation of THCA-A into hair via blood, sebum or sweat, a volunteer ingested 50 mg THCA-A daily over a 30 day period (c_max_ of THCA-A in serum was 2,120 ng/ml^16^, oral bioavailability of THCA-A: approximately 41%^17^). Despite a relatively high dose of 50 mg THCA-A per day (a heavy cannabis user may take up doses of several hundred mg of total THC daily, and the proportion of THCA-A in cannabis smoke was found to be less than 1% by weight^18^), no THCA-A could be detected in any of the segmented hair samples obtained during the study. In accordance with the hair analysis results, no THCA-A could be detected in any of the sebum/sweat samples either.

### Oral intake of dronabinol

**Figure 2:**
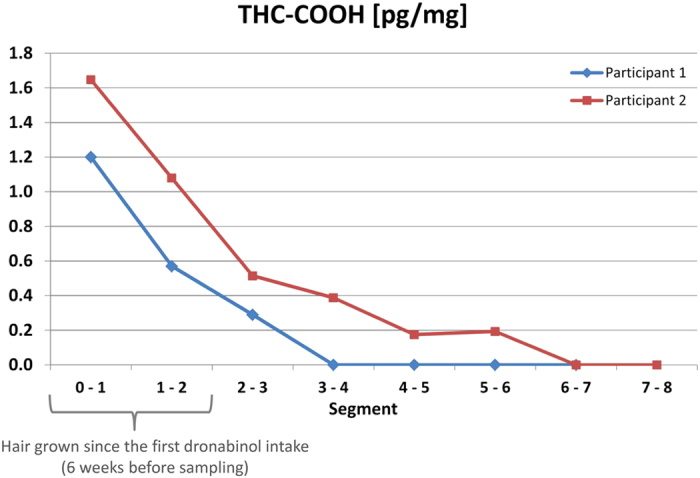
Distribution of THC-COOH along the hair shaft after dronabinol intake. 11-*nor*-9-carboxy-∆9-tetrahydrocannabinol (THC-COOH) concentrations determined in the segmented head hair samples of two study participants obtained two weeks after the last intake of dronabinol (3 × 2.5 mg daily for 30 days).

**Figure 3:**
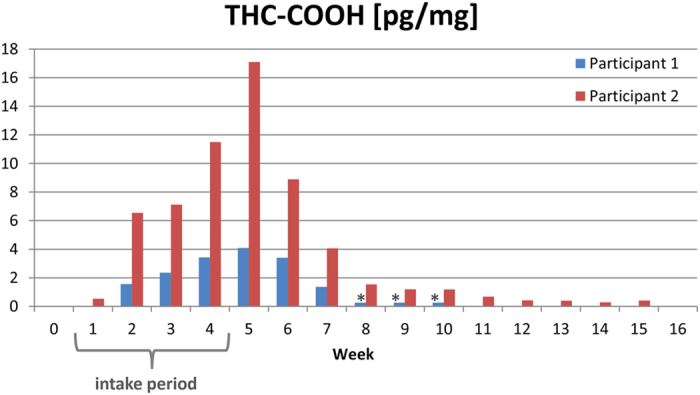
THC-COOH concentration in beard hair after dronabinol intake. 11-*nor*-9-carboxy-∆9-tetrahydrocannabinol (THC-COOH) concentrations determined in the beard hair samples of two study participants before and after the intake of dronabinol (3 × 2.5 mg daily for 30 days). *For participant 1 only one sample was obtained covering weeks 8–10.

In a second study, consisting of repeated oral intake of dronabinol (THC) by two volunteers over a 30 day period (2.5 mg, three times per day), the extent of THC incorporation via the bloodstream into hair was evaluated. No THC was detected at any time of sampling in all the head hair, beard hair or body hair samples (limit of detection: 1 pg/mg). From multiple serum samples taken within 8 hours (dosing interval) the estimated AUC_0→24 h_ (THC) of the two participants ranged from 740–1,300 μg/L * min (n = 3 for each participant). Maximum serum concentrations of THC-COOH were 18 ng/mL (participant 1) and 40 ng/mL (participant 2), respectively (see Supplementary Tables S3 and S4 online). Considering the individual head hair growth rates (1.3 cm per month for both participants), THC-COOH was also detected in segments correlating to a time period located up to 2.3–3.1 months before the start of the THC intake (Fig. 2: Participant 2 showed THC-COOH positive results up to the segment 5–6 cm collected six weeks after the first intake, for participant 1 the most distal positive segment was 2–3 cm corresponding to maximum 3–4 weeks before start of THC intake; for full data see Supplementary Table S1 online). Analysing sebum/sweat samples of both participants revealed THC-COOH amounts of 4.3 to 82 pg/cm^2^ per day (Table 1). Analysis of hair samples from alternative sampling sites tended to show relatively high concentrations in beard, pubic and axillary hair (see Supplementary Table S2 online). In beard hair samples, THC-COOH could be detected up to 11 weeks after the last THC intake (Fig. 3).

## Discussion

The results strongly suggest that THCA-A is not incorporated into hair through the bloodstream or via sebum/sweat to a relevant extent. Although this was tested only in one individual, the daily dose of THCA-A was at least an order of magnitude higher than expected in excessive cannabis smokers. Therefore, the THCA-A detected in forensic hair samples (concentration range in hair samples of cannabis consumers: 46–4,700 pg/mg^13^) can only be explained by external contamination via handling of cannabis material^15^.

The incorporation rate of THC via the bloodstream into the hair seems to be negligible low as no THC could be detected in the hair samples of the participants after systemic dronabinol uptake. It follows from Fick’s law that the amount of analyte incorporated into hair should be proportional to the area under the analyte serum concentration over time curve (AUC). Given that the THC AUC_0→24 h_ of the two participants was only less than five times lower than the AUC range found in the literature for occasional cannabis smokers after a single consumption (780–6,300 μg/L * min)^19^,^20^, it is obvious that also no relevant incorporation through the bloodstream into hair is expected to occur in cannabis users, and THC detected in forensic hair samples does originate from external sources. To reach THC concentrations of 50 pg/mg (cut-off recommended by the Society of Hair Testing^21^) through incorporation via the bloodstream would require consumption of extremely high amounts of THC, which would certainly be associated with a several-fold higher amount of THC incorporated through contamination routes (cannabis smoke exposition and/or transfer by contaminated fingers). Consequently, THC findings in hair cannot be regarded as a proof of cannabis consumption. At the same time, oral uptake of THC or cannabis products does not necessarily lead to positive THC hair findings, which can be of interest in abstinence control.

Furthermore, the detection of THC-COOH in hair segments did not correlate to the period of THC intake and the presence of THC-COOH in sebum/sweat implicates a relevant contribution to the THC-COOH findings in hair samples by diffusion of the analyte from sebum into the hair matrix. The marked variations in the THC-COOH concentrations between body regions may be explained by differences in the physiology (e.g. presence of apocrine sweat glands in the axillary and pubic region), sampling particularities (e.g. regular shaving of beard hair vs. sampling of hair strands) and a possible transfer of the analyte due to contamination of hair with urine (pubic region). The fact, that THC-COOH was detectable up to 11 weeks past the intake period in beard hair further underlines a relevant incorporation via secretion of sebum which shows a physiological time shift, or by diffusion from surrounding tissues^2^.

At first glance, differentiation of the route of THC-COOH incorporation into hair seems irrelevant as long as positive THC-COOH findings in hair require THC uptake by the individual under investigation. However, considering the presence of THC-COOH in sebum/sweat, a transfer to other persons’ hair is possible. This is particularly true for young children or partners of cannabis consumers (close body contact, sleeping on the same pillow etc.). Comparing the maximum serum THC-COOH concentrations detected in persons massively exposed to cannabis smoke in a ‘coffee shop’ (0.5–1.7 ng/mL)^22^ to the maximum serum concentrations determined in our study (18 and 40 ng/mL), it seems very unlikely that passive smoke exposition can lead to similar THC-COOH concentrations in hair as chronic active consumption does. However, THC-COOH can be detected in hair of young children (age: <2 years)^23^ in concentrations similar to the concentrations detected in the hair after oral dronabinol intake. Therefore, it seems much more plausible that THC-COOH is transferred to the children’s hair by close contact to the cannabis consumers in the family context rather than by systemic uptake after exposition to cannabis smoke.

### Limitations of the study

For the oral intake of THCA-A only one individual was tested. Although the extraordinary high serum concentrations reached should compensate for this, physiological characteristics of the individual may have led to THCA-A not being incorporated into hair to a measurable extent. In the study with oral intake of dronabinol a relatively low dose of THC was used, which may reflect THC uptake of moderate cannabis smokers, but not of heavy users. Therefore, measurable incorporation of THC from the blood stream cannot be excluded in the case of heavy cannabis smoking. Due to oral administration (slow resorption, first-pass effect) the maximum THC serum concentrations were lower than the maximum concentrations generally reached after smoking. Although – following from Fick’s law – incorporation should be proportional to the AUC, the diffusion coefficient may vary with the gradient. High concentration gradients as observed directly after smoking might therefore lead to a more efficient incorporation of THC. Furthermore, the number of individuals tested in this study was low (n = 2) and pharmacokinetic particularities may affect the generalizability of the findings.

## Conclusions

Knowing the main routes of cannabinoid incorporation into human hair, any interpretation of varying concentrations along the hair shaft in terms of time-resolved patterns of use may lead to false conclusions. Cases with high THC or THC-COOH concentrations in proximal hair segments are in particular critical as they may be interpreted as a recent increase of cannabis consumption. Not over-interpreting THC or THC-COOH findings in hair is of utmost importance in child protection cases, but also in the context of work place drug testing and any forensic application. Practitioners who work with results of hair analysis should be aware of these limitations and the severe consequences false conclusions could entail.

Although the results of our study cannot be transferred directly to other cannabinoids or other types of illicit drugs (in particular to less lipophilic and non-acidic compounds) the proportion of drugs incorporated into hair via the bloodstream is largely unknown and should be the focus of further research.

## Methods

### Ethical approval

The study protocol was approved by the Ethics Committee of the University of Freiburg, Germany (EK-Freiburg 98/14), and the Federal Opium Agency (BfArM, Bonn, Germany) granted a permit for the intake of dronabinol. The study was registered in a World Health Organization primary register (German Clinical Trials Register; DRKS00006148, registered: 8^th^ May 2014), and was conducted at the Institute of Forensic Medicine Freiburg, Germany, in accordance with the Declaration of Helsinki Principles and subsequent amendments. Written informed consent was obtained from each participant. Volunteers were recruited from the personal environment of the authors and affirmed that they neither consumed cannabis nor were exposed to cannabis via peers or family members prior to and during the study.

#### Oral intake of THCA-A

One male volunteer orally ingested 50 mg THCA-A daily over a 30 day period. Hair samples (head, chest, pubic, axillary and leg hair) were collected prior to the intake period, and then on a weekly basis until three weeks after the last intake. The segmented hair samples (1 cm segments) were analysed for THCA-A applying a fully validated LC-MS/MS method^24^. See Supplementary Material for details.

#### Oral intake of dronabinol

Two male participants orally ingested 2.5 mg dronabinol (THC) three times daily over a 30 day period. Hair samples (head, beard and body hair) were collected prior to the intake period, and then on a regular basis until several weeks after the last intake. Apart from hair samples, combined sebum/sweat samples were collected using Sebutapes®. All hair samples were analysed for THC and THC-COOH after alkaline hydrolysis applying a fully validated LC-MS^3^ method on a Shimadzu Nexera 2 UHPLC coupled to an ABSciex QTRAP 5500 linear ion-trap mass spectrometer. See Supplementary Material for details.

## Additional Information

**How to cite this article**: Moosmann, B. *et al.* Finding cannabinoids in hair does not prove cannabis consumption. *Sci. Rep.* **5**, 14906; doi: 10.1038/srep14906 (2015).

## Supplementary Material

Supplemental Data

## Figures and Tables

**Figure 1:**
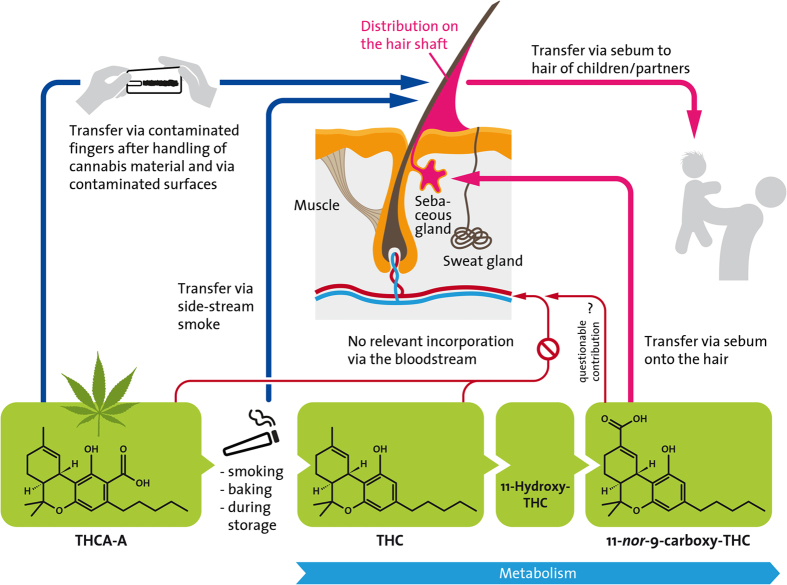
Potential incorporation pathways of cannabinoids into human hair. Incorporation of ∆9-tetrahydrocannabinolic acid A (THCA-A), ∆9-tetrahydrocannabinol (THC), and its metabolite 11-*nor*-9-carboxy-THC (THC-COOH) into human hair can occur in the hair bulb via the bloodstream, by diffusion from sweat or sebum into the hair shaft, or by external contamination (e.g. contaminated fingers or side-stream smoke). The main metabolic pathway of THC and the molecular structures of the respective analytes are also given.

**Table 1:** THC-COOH concentrations in sebum/sweat samples.

Participant 1		Participant 2	
Sampling time	THC-COOH [pg/cm^2^/day]^a^	Sampling time	THC-COOH [pg/cm^2^/day]^a^
Day 0	n.d.	Day 0	n.d.
Day 3/4	56	Day 1/2	58
Day 6/7	19	Day 2/3	69
Day 9/10	13	Day 5/6	47
Day 10/11	4.5	Day 7/8	41
Day 22/23	6.9	Day 8/9	82
Day 26/27	6.2	Day 10/11	31
Day 27/28	8.0	Day 13/14	57
Day 30/31	5.6	Day 21/22	42
1 day after last intake	7.2	Day 30/31	36
2 days after last intake	4.3	5 days after last intake	33
3 days after last intake	27	6 days after last intake	6.2
5 days after last intake	n.d.	7 days after last intake	32
6 days after last intake	n.d.	8 days after last intake	15
7 days after last intake	n.d.	11 days after last intake	35
9 days after last intake	4.5	12 days after last intake	24
10 days after last intake	n.d.	13 days after last intake	26
13 days after last intake	12	14 days after last intake	39
		18 days after last intake	11
		25 days after last intake	46
		27 days after last intake	n.d.

11-*nor*-9-carboxy-∆9-tetrahydrocannabinol (THC-COOH) concentrations determined in the sebum/sweat samples of the participants collected prior to the dronabinol intake period (3 × 2.5 mg daily for 30 days), and then on a regular basis until several weeks after the last intake. The samples were collected by using Sebutape^®^ patches which were placed on the forehead overnight.

n.d.: not detected (limit of detection 0.8 pg/cm^2^).

^a^Sebum/sweat concentration were normalised to day intervals for better comparability.
