# Supplementary material for: Finding cannabinoids in hair does not prove cannabis consumption
Source: Sci Rep. 2015 Oct 7;5:14906. doi: 10.1038/srep14906 (PMC4595642; doi:10.1038/srep14906)
Supplement: Supplemental Data [file srep14906-s1.doc]

**Finding cannabinoids in hair does not prove cannabis consumption**

Bjoern Moosmann, Nadine Roth and Volker Auwärter

**Supplemental Data**

Materials and Methods

Supplementary Tables S1-S5

References 25, 26

25. Roth, N., Wohlfarth, A., Müller, M. & Auwärter, V. Regioselective synthesis of isotopically labeled D9-tetrahydrocannabinolic acid A (THCA-A-D3) by reaction of D9-tetrahydrocannabinol-D3 with magnesium methyl carbonate. *Forensic Sci. Int.* **222**, 368-372 (2012).

26. Wohlfarth, A., Mahler, H. & Auwärter, V. Rapid isolation procedure for D9-tetrahydrocannabinolic acid A (THCA) from Cannabis sativa using two flash chromatography systems. *J. Chromatogr. B* **879**, 3059-3064 (2011).

**Methods**

**Materials**

Acetonitrile, methanol (MeOH) (both gradient grade) and sodium hydroxide were obtained from J.T. Baker (Deventer, the Netherlands). Formic acid (ROTIPURAN® ≥98%, p.a.) and petroleum ether (ROTIPURAN® 40‑60°°C) were purchased from Carl Roth (Karlsruhe, Germany), *tert*-butyl methyl ether, acetic acid, glacial acetic acid, acetone (p.a., ACS) and ethyl acetate from Sigma Aldrich (Steinheim, Germany). *N*-methyl-*N*-trimethylsilyltrifluoroacetamide was from Macherey-Nagel (Düren, Germany) and lecithin (egg phosphatidylcholine) from Lipoid (Ludwigshafen, Germany). THC, THC-COOH, THC‑D_3_ and THC-COOH-D_3_ were obtained from Cerilliant (Round Rock, TX, USA). THCA‑A was purchased from Lipomed (Arlesheim, Switzerland) and THCA‑A‑D_3_ was synthesised according to Roth *et al.*^25^*.* The THCA‑A used for the oral intake study was isolated from cannabis sativa following the protocol published by Wohlfarth *et al.*^26^*.* Deionized water was prepared using a cartridge deionizer from Memtech (Moorenweis, Germany). Blank hair was provided by volunteers and tested for cannabinoids prior to use.

**Oral intake of THCA-A**

One male volunteer orally ingested 50 mg THCA-A in sesame oil in a gelatine capsule daily over a 30 day period. Head hair samples were collected prior to the intake period, and then on a weekly basis until three weeks after the last intake. At the last sampling date four additional samples from various regions of the head as well as armpit, chest, leg and pubic hair were collected. Furthermore, combined sebum and sweat samples were collected for up to one month post intake by using Sebutapes® (Cuderm Corp. Dallas, TX, USA) which were placed on the forehead overnight. Hair samples were washed, extracted and analyzed for THCA‑A applying a fully validated LC-MS/MS method^24^. For head hair samples, only the proximal 3 cm of the hair shaft, divided into 1 cm segments, were analyzed. Since hair matrix can vary greatly between individuals, all the blank hair used for the calibrators was from the same volunteer to substantiate the results especially in terms of occurring matrix effects. Furthermore, by spiking various blank samples of this particular volunteer with concentrations (0.1 – 2.5 pg/mg) below the validated limit of detection, the specific limit of detection of 1 pg/mg for this author’s hair matrix was assessed. Determination of THCA-A in sebum/sweat samples was carried out by placing the worn Sebutape® with the active site facing downwards on a ‘headspace’ vial filled with 2 mL methanol and 20 µL internal standard solution (3 ng THCA-A-D_3_). The vials were tightly closed with a septum and an aluminium crimp cap and shaken upside down for 30 min. The extract was transferred into an HPLC vial evaporated to dryness and reconstituted in 100 µL solvent (acetonitrile + 0.1 % HCOOH + 0.25% lecithin (w/v)). Finally, the samples were analyzed applying LC-MS/MS analysis with the identical method as described for the hair samples above.

**Oral intake of dronabinol**

Two male participants orally ingested 2.5 mg dronabinol (THC) (THC Pharm, Frankfurt, Germany) three times daily over a 30 day period. The capsules were taken under direct observation of a study supervisor and completed as scheduled. Head hair samples were collected prior to the intake period, and then on a regular basis until several weeks after the last intake (for sampling schedule see Table S1). Furthermore, beard hair samples were collected on a weekly basis up to 12 weeks after the last intake and hair samples from numerous body regions two weeks after the last dose (for details see Table S2). Hair growth rates of the head hair were assessed by multiple sampling and were 1.3 cm per month for both participants in the posterior vertex region. Apart from hair samples, combined sebum/sweat samples were collected using Sebutapes® (Cuderm Corp. Dallas, TX, USA) worn on the forehead overnight (for details see Table 1). In addition, serum samples were collected on a regular basis (for details see Tables S3 and S4). Head hair samples were cut into 1 cm segments prior to analysis. Beard and body hair samples were processed without segmentation. All the hair samples were analysed using LC-MS^3^ analysis after alkaline hydrolysis. In brief, 20 µL internal standard solution (0.2 ng THC-COOH-D_3_ and 2 ng THC-D_3_) was added to 20 mg of hair and the hair matrix was digested in 1 mL 1 M sodium hydroxide at 95 °C for 10 min. Subsequently, 0.5 mL 20 % acetic acid as well as 2 mL hexane/EE (9:1 v/v) were added and the mixture shaken for 5 min, and centrifuged for 5 min. Afterwards, the supernatant was transferred into a separate vial, evaporated to dryness and reconstituted in 50 µL mobile phase B (MeOH/0.1 % HCOOH 95:5 (v/v)). Finally, 20 µL were injected into the LC-MS^3^ system consisting of a Nexera 2 UHPLC (Shimadzu, Duisburg, Germany) coupled to a QTRAP 5500 linear ion-trap mass spectrometer (ABSciex, Darmstadt, Germany). For separation gradient elution was applied using 0.1 % HCOOH (A) and MeOH/ A (95:5 v/v) (B) on a Kinetex XB-C18 column (100 mm x 2.1 mm, 2.6 µm) with a C18 guard column (2 mm x 2.1 mm) (both Phenomenex, Aschaffenburg, Germany). The gradient was started with 10 % B for 1 min, increased to 60 % B in 2 min and further increased to 100 % B in 5 min followed by a 2 min hold at 100 % B. Starting conditions were restored within 0.1 min and the system was allowed to re-equilibrate for 2.9 min. Flow rate was set at 0.8 mL/min and the column oven was heated to 50 °C. All the samples were stored in the autosampler at 25 °C prior to analysis. MS^3^ acquisition was carried out in two periods (0 – 6.4 min & 6.4 – 13 min) and the parameters are given in Supplementary Table S5. The mass spectrometer was operated using negative electrospray ionization (ESI) with an ion source voltage of -4500 V in the first period and in positive ESI with an ion source voltage of +4500 V in the second period. The ion source temperature was set at 600 °C; the linear ion trap fill time was set to 250 ms for both analytes and to 20 ms for the respective deuterated standards, the scan rate was set to 10,000 Da/s. Limit of detection was 0.1 pg/mg for THC‑COOH and 1 pg/mg for THC.

Determination of THC and THC‑COOH in sebum samples were carried out by placing the worn Sebutape® with the active site facing downwards on a ‘headspace’ vial filled with 2 mL methanol and 20 µL internal standard solution (0.2 ng THC-COOH-D_3_ and 2 ng THC-D_3_). The vials were tightly closed with a septum and an aluminium crimp cap and shaken upside down for 30 min. The extract was transferred into an HPLC vial evaporated to dryness and reconstituted in 50 µL mobile phase B (MeOH/0.1 % HCOOH 95:5 (v/v)). Finally, the samples were analysed applying LC-MS^3^ analysis with the identical method as described for the hair samples above.

Determination of THC and THC‑COOH in serum and urine was carried out according to the accredited routine procedure for the determination of cannabinoids in forensic samples. Twenty five microliter internal standard solution (5 ng THC-D_3_ and 25 ng THC-COOH-D_3_) were added to 1 mL serum. The samples were diluted with 0.1 M acetic acid up to a volume of 3 mL prior to automated solid‑phase extraction using an GX-274 ASPEC (Gilson, Limburg-Offheim, Germany) equipped with 3 mL Chromabond C_18_ 500 mg cartridges (Macherey-Nagel, Düren, Germany). The extraction program consisted of the following steps: conditioning: 2 mL MeOH and 2 mL 0.1 M acetic acid; sample loading; washing: 1 mL acetic acid and 1 mL 70 % ACN; drying for 1 min; eluting: 1.5 mL ACN. After evaporation to dryness 25 µL MSTFA and 25 µL ethyl acetate were added to the residue and the sample was derivatised for 45 min at 90 °C. After cooling, 1 µL were injected splitless into the GC-MS system consisting of a 6890 series GC system, a 5973 series mass selective detector, a 7683 B series injector and Chemstation G1701GA version D.03.00.611 software (Agilent, Waldbronn, Germany). The GC parameters and MS conditions were as follows: column: HP5-MS capillary (30 x 0.25 mm i.d., 0.25 µm film thickness; Agilent); injection port temperature: 250 °C; carrier gas: helium; flow rate: 1.5 mL/min; oven temperature: 140 °C for 2 min, ramped to 200 °C at 60 °C/min, ramped to 230 °C at 2.5 °C/min, ramped to 310 °C at 60 °C/min, held 310 °C for 4 min; ion source temperature: 230 °C; electron impact (EI) mode; ionization energy: 70 eV. Quantification of the trimethylsilyl derivatives was performed in SIM mode using the following fragment ions (m/z; quantifiers bold): THC-D_3_ **389**, 374, 306, THC **386**, 371, 303, THC-COOH-D_3_ **374**, 491, 476, THC-COOH **371**, 488, 473.

**Table S1: THC-COOH concentrations in head hair samples.** 11-*nor*-9-carboxy-∆9-tetrahydrocannabinol (THC-COOH) concentrations determined in the 1­cm segmented head hair samples of two study participants before and after the intake of dronabinol (3 x 2.5 mg daily for 30 days).

| **Participant 1** |  |  |  | **Participant 2** |  |  |
| --- | --- | --- | --- | --- | --- | --- |
| Sampling time | Segment(s) | THC-COOH [pg/mg] |  | Sampling time | Segment(s) | THC-COOH [pg/mg] |
| Before the study | all (5 cm) | n.d. |  | Before the study | all (6 cm) | n.d. |
| End of week 1 | all (5 cm) | n.d. |  | End of week 2 | 0 - 1 cm | 0.48 |
| End of week 2 | all (5.5 cm) | n.d. |  |  | 1 - 2 cm | 0.26 |
| End of week 3 | 0 - 1 cm | 0.32 |  |  | 2 - 3 cm | 0.27 |
|  | rest (1-6 cm) | n.d. |  |  | rest (3-6.5 cm) | n.d. |
| End of intake period | 0 - 1 cm | 1.2 |  | End of week 3 | 0 - 1 cm | 0.91 |
|  | 1 - 2 cm | 0.52 |  |  | 1 - 2 cm | 0.33 |
|  | rest (2-6 cm) | n.d. |  |  | 2 - 3 cm | 0.32 |
| 1 week after last intake | 0 - 1 cm | 0.42 |  |  | rest (3-7 cm) | n.d. |
|  | 1 - 2 cm | 0.45 |  | End of intake period | 0 - 1 cm | 1.1 |
|  | rest (2-6.5 cm) | n.d. |  |  | 1 - 2 cm | 0.40 |
| 2 weeks after last intake | 0 - 1 cm | 1.2 |  |  | 2 - 3 cm | 0.26 |
|  | 1 - 2 cm | 0.57 |  |  | rest (3-7.5 cm) | n.d. |
|  | 2 - 3 cm | 0.29 |  | 2 weeks after last intake | 0 - 1 cm | 1.70 |
|  | rest (3-7 cm) | n.d. |  |  | 1 - 2 cm | 1.10 |
|  |  |  |  |  | 2 - 3 cm | 0.52 |
|  |  |  |  |  | 3 - 4 cm | 0.39 |
|  |  |  |  |  | 4 - 5 cm | 0.18 |
|  |  |  |  |  | 5 - 6 cm | 0.19 |
|  |  |  |  |  | rest (6-8 cm) | n.d. |

n.d.: not detected (limit of detection 0.1 pg/mg)

**Table S2: THC-COOH concentrations in hair samples collected from alternative sampling sites.** 11-*nor*-9-carboxy-∆9-tetrahydrocannabinol (THC-COOH) concentrations determined in the hair samples obtained from alternative sampling sites of two study participants before and after the intake of dronabinol (3 x 2.5 mg daily for 30 days).

| **Participant 1** |  |  |
| --- | --- | --- |
| Sampling time | Sampling site | THC-COOH [pg/mg] |
| Before the study | Pubic region | n.d. |
|  | Armpit | n.d. |
| End of intake period | Pubic region | 1.4 |
|  | Armpit | 0.33 |
|  | Back | 0.75 |
|  | Lower leg | 0.22 |
|  | Upper leg | 0.44 |
|  | Chest | 0.14 |
|  | Arm | 0.29 |
| 2 weeks after last intake | Pubic region | 1.2 |
|  | Armpit left | 0.36 |
|  | Armpit right | 0.53 |
|  | Neck | 0.64 |
|  | Abdominal | 0.20 |
|  | Chest | 0.16 |
|  | Knee | 0.30 |
|  | Lower leg left | 0.26 |
|  | Lower leg right | 0.17 |
|  | Upper leg | 0.53 |
|  | Buttocks | 0.32 |
|  | Arm | 0.16 |
|  |  |  |
|  |  |  |
| **Participant 2** |  |  |
| Sampling time | Sampling site | THC-COOH [pg/mg] |
| Before the study | Pubic region | n.d. |
|  | Armpit | n.d. |
| End of intake period | Pubic region | 4.7 |
| 2 weeks after last intake | Pubic region | 3.3 |
|  | Armpit left | 5.6 |
|  | Armpit right | 4.9 |
|  | Abdominal | 0.97 |
|  | Chest | 1.57 |
|  | Knee | 0.64 |
|  | Lower leg left | 0.64 |
|  | Lower leg right | 0.65 |
|  | Upper leg | 0.49 |
|  | Buttocks | 3.4 |
|  | Arm | 1.1 |

n.d.: not detected (limit of detection 0.1 pg/mg)

**Table S3:** **THC and THC-COOH serum concentrations of participant 1.** ∆9-tetrahydrocannabinol (THC) and 11-*nor*-9-carboxy-THC (THC-COOH) concentrations determined in serum samples obtained from participant 1 after the intake of dronabinol (steady state conditions).

| Sampling time after intake [min] | THC [ng/mL] | THC-COOH [ng/mL] |  |
| --- | --- | --- | --- |
| 0 | 0.2 | 13 |  |
| 30 | 0.23 | 12 |  |
| 60 | 0.24 | 13 |  |
| 90 | 0.21 | 10 |  |
| 120 | 0.2 | 10 |  |
| 135 | 0.19 | 9.2 |  |
| 150 | 0.28 | 10 |  |
| 165 | 0.7 | 10 |  |
| 180 | 0.99 | 10 |  |
| 195 | 0.74 | 9.6 |  |
| 210 | 0.53 | 10 |  |
| 225 | 0.5 | 8.8 |  |
| 240 | 0.73 | 11 |  |
| 270 | 1.1 | 18 |  |
| 300 | 1.5 | 16 |  |
| 330 | 0.46 | 9.7 |  |
| 360 | 0.41 | 9.5 |  |
| 420 | 0.25 | 7.7 |  |
| 480 | 0.2 | 8.0 |  |

**Table S4**: **THC and THC-COOH serum concentrations of participant 2.** ∆9-tetrahydrocannabinol (THC) and 11-*nor*-9-carboxy-THC (THC-COOH) concentrations determined in serum samples obtained from participant 2 after the intake of dronabinol (steady state conditions).

| Sampling time after intake [min] | THC [ng/mL] | THC-COOH [ng/mL] |
| --- | --- | --- |
| 0 | 0.34 | 28 |
| 20 | 0.33 | 32 |
| 26 | 0.31 | 31 |
| 35 | 0.31 | 28 |
| 45 | 0.27 | 28 |
| 55 | 0.29 | 28 |
| 65 | 0.27 | 28 |
| 80 | 0.34 | 31 |
| 100 | 1.7 | 33 |
| 125 | 2.8 | 40 |
| 155 | 1.4 | 37 |
| 215 | 0.97 | 33 |
| 305 | 1.7 | 39 |
| 430 | 0.53 | 39 |
| 480 | 0.37 | 33 |

**Table S5: Mass spectrometric parameters for the analysis of THC and THC-COOH in hair and sebum.** Summary of parent, secondary precursor and product ions as well as mass spectrometric parameters for ∆9-tetrahydrocannabinol (THC), 11-*nor*-9-carboxy-THC (THC-COOH), and their respective deuterated analogues.

| **Analyte** | **Parent ion [m/z]** | **Secondary precursor ion [m/z]** | **MS^3^ range for quantification [m/z])** | **DP [V]** | **CE [V]** | **AF2** | **Excitation time [ms]** |
| --- | --- | --- | --- | --- | --- | --- | --- |
| *Period 1 (0 - 6.4 min)* | |  |  |  |  |  |  |
| THC-COOH | 343.3 | 299.2 | 244.7 - 245.7 | -140 | -31 | 0.09 | 30 |
| THC-COOH-D3 | 346.3 | 302.2 | 243 - 253 | -80 | -30 | 0.09 | 20 |
|  |  |  |  |  |  |  |  |
| *Period 2 (6.4 - 13 min)* | |  |  |  |  |  |  |
| THC | 315 | 193 | 118 - 128 | 60 | 26 | 0.1 | 25 |
| THC-D3 | 318 | 196 | 121 - 131 | 60 | 35 | 0.1 | 25 |

DP: Declustering potential

CE: Collision energy

AF2: Excitation energy
